# CD8^+^ T cells from HLA-B*57 elite suppressors effectively suppress replication of HIV-1 escape mutants

**DOI:** 10.1186/1742-4690-10-152

**Published:** 2013-12-12

**Authors:** Christopher W Pohlmeyer, Robert W Buckheit, Robert F Siliciano, Joel N Blankson

**Affiliations:** 1 Department of Medicine, Johns Hopkins University School of Medicine, 733 N. Broadway, BRB 880, Baltimore, MD 21205, USA; 2 Howard Hughes Medical Institute, Johns Hopkins University School of Medicine, 733 N. Broadway, Baltimore, MD 21205, USA; 3 Current address: Virus-Cell Interaction Section, HIV Drug Resistance Program, National Cancer Institute at Frederick, Frederick, MD 21702-1201, USA

**Keywords:** HIV-1, Elite suppressor, Elite controller, Escape mutation, CTL

## Abstract

**Background:**

Elite Controllers or Suppressors (ES) are HIV-1 positive individuals who maintain plasma viral loads below the limit of detection of standard clinical assays without antiretroviral therapy. Multiple lines of evidence suggest that the control of viral replication in these patients is due to a strong and specific cytotoxic T lymphocyte (CTL) response. The ability of CD8^+^ T cells to control HIV-1 replication is believed to be impaired by the development of escape mutations. Surprisingly, viruses amplified from the plasma of ES have been shown to contain multiple escape mutations, and it is not clear how immunologic control is maintained in the face of virologic escape.

**Results:**

We investigated the effect of escape mutations within HLA*B-57^-^restricted Gag epitopes on the CD8^+^ T cell mediated suppression of HIV-1 replication. Using site directed mutagenesis, we constructed six NL4-3 based viruses with canonical escape mutations in one to three HLA*B-57-restricted Gag epitopes. Interestingly, similar levels of CTL-mediated suppression of replication in autologous primary CD4^+^ T cells were observed for all of the escape mutants. Intracellular cytokine staining was performed in order to determine the mechanisms involved in the suppression of the escape variants. While low baseline CD8^+^ T cells responses to wild type and escape variant peptides were seen, stimulation of PBMC with either wild type or escape variant peptides resulted in increased IFN-γ and perforin expression.

**Conclusions:**

These data presented demonstrate that CD8^+^ T cells from ES are capable of suppressing replication of virus harboring escape mutations in HLA-B*57-restricted Gag epitopes. Additionally, our data suggest that ES CD8^+^ T cells are capable of generating effective *de novo* responses to escape mutants.

## Background

In primary HIV-1 infection, vigorous viral replication results in plasma virus levels as high as one million copies/mL of HIV-1 RNA. As cellular immune responses develop, plasma virus levels decrease, and a viral set point is established. A subset of HIV-1-infected individuals known as Elite Controllers or Suppressors (ES), maintain a viral set point below the limit of detection of standard clinical assays (<50 copies of HIV-1 RNA/mL of blood [1-4]). Early studies suggested that some long-term non-progressors (LTNPs) and ES are infected with defective viruses [5,6]. In contrast, more recent studies have determined that many ES are infected with viruses that are fully replication competent, suggesting that host factors rather than infection by defective virus are responsible for ES status [7-11].

Some HLA alleles affect disease progression, including HLA-B*27, HLA-B*51, HLA-B*57/58, and HLA-B*35 [12,13]. Multiple cohort studies have demonstrated that the HLA-B*57 allele is overrepresented in ES [14-20]. This finding has been confirmed by multiple GWAS studies [21-26]. However, the majority of HIV-1-infected HLA-B*57^+^ patients develop progressive disease, and are thus termed chronic progressors (CPs) [14]. The protection conferred by HLA-B*57 and HLA-B*27 is thought to be mediated by effective CD8^+^ T cell responses against conserved immunodomniant epitopes that are presented by MHC class I proteins [18,27-32]. Comparison studies of HLA-B*57^+^ ES and CPs have provided some details about the elite suppressor phenotype. Stimulation of bulk peripheral blood mononuclear cells (PBMCs) with Gag peptides induces greater proliferation as well as more robust granzymes A/B and perforin expression in CD8^+^ T cells from HLA-B*57^+^ ES compared to HLA-B*57^+^ CPs [18].

Some nonsynonymous mutations in epitopes enable the virus to escape from the cytotoxic T lymphocyte (CTL) responses. The role these escape mutations play in determining protection versus progression in HLA-B*57 positive patients is controversial. A correlation between the number of HLA-B*57 Gag epitopes and the level of viremia was observed in a cohort of HLA-B*5703 positive patients with Clade C HIV-1 infection [33]. In another study, the development of escape mutations was temporally associated with virologic breakthrough in a patient who had maintained undetectable viral loads for a year after infection [34]. In contrast, studies of early infection in other HLA-B*57/5801 positive patients have not found a correlation between the accumulation of escape mutations and virologic breakthrough [35,36]. Furthermore, one study found no difference in the frequency of escape mutations in HLA-B*57-restricted epitopes in proviral clones amplified from ES when compared to CPs [37]; other studies found that while escape mutations were largely absent from provirus amplified from CD4^+^ T cells of ES, virus amplified from the plasma of the same subjects contained a high frequency of escape mutations [38,39]. In this study, we sought to explain how ES maintain undetectable levels of plasma virus despite the presence of circulating HIV-1 isolates that contain numerous escape mutations. Specifically, we asked whether CD8^+^ T cells from these patients were capable of inhibiting the viral replication of engineered escape mutants. We demonstrate that ES can inhibit the replication of escape variant HIV-1 and suggest that these patients are capable of generating protective *de novo* responses against the escape mutant variants. This work has implications for the design of therapeutic T cell vaccines to prevent the progression of HIV-1 disease.

## Results

### Effect of escape mutations in HLA-B*57-restricted Gag epitopes on viral fitness

**Figure 1:**
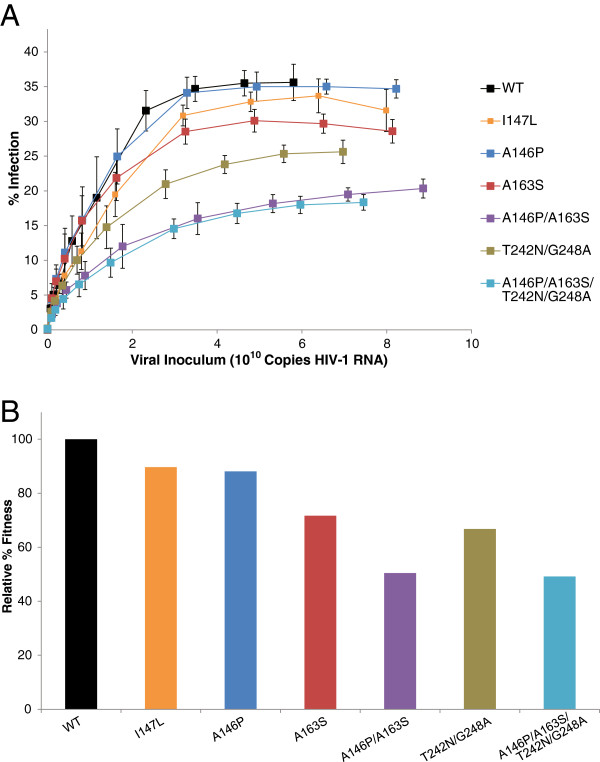
Fitness cost of canonical HLA-B*57 escape mutations. A. Average infection by seven NL4-3 escape variants in uninfected individuals. Viral inoculum was quantified by relative qPCR. 10^5^ CD4^+^ T cells were infected in a 96-well plate in triplicate. Infection was determined by GFP expression by flow cytometry. Wild type NL4-3 (black) showed the highest level of infection, while NL4-3 escape mutant variants showed reduced maximal infection (I147L, orange; A146P, navy; A163S, red; A146P/A163S, purple; T242N/G248A, brown; A146P/A163S/T242N/G248A, teal). Error bars represent SEM. n = 4. **B**. Maximal infection of each escape variant is compared relative to wild type NL4-3 virus.

The effect of several escape mutations on viral fitness has been explored *in vivo* and *in vitro*[40-45]. We focused on escape mutations in the three HLA-B*5703 restricted Gag epitopes: IW9 (Gag 147–155), KF11 (Gag 162–172), and TW10 (Gag 240–249). We have previously demonstrated that HLA-B*5703 positive ES in our cohort do not target the fourth HLA-B*57-restricted Gag epitope QW9 (Gag 308–316) [38] and therefore we did not include this epitope in our analysis. To address the fitness cost of these escape mutations, we introduced a series of mutations into the reference isolate NL4-3 and generated GFP-expressing HIV-1 pseudoviruses carrying these mutations. Virus concentrations were measured in triplicates by RT-PCR. We then infected PHA-activated CD4^+^ T cells from four seronegative healthy donors using a 2 log range of virus inoculums. Infection curves for the seven viruses are shown in Figure 1A.

Infectivity relative to the reference clone NL4-3 was taken as a measure of fitness. Infectivity curves plateaued at different points for different mutants. A best fitting curve was generated with GraphPad to calculate a nonlinear least squares regression model and was used to determine the theoretical maximal infection. Additional file 1: Figure S1 shows the best fit curve for each of the assayed viruses. This theoretical maximal infection was used to determine the relative percentage of maximal infection relative to the reference clone NL4-3 (Figure 1B). The I147L and A146P mutations, which are found in the IW9 epitope of p24, each had a minimal effect on viral fitness with calculated fitness levels of 88 percent and 90 percent respectively compared to unmutated NL4-3. The A163S mutation (72 percent relative fitness) had a larger effect on fitness consistent with a prior study that showed that mutations in this epitope in Clade C virus had a major impact on replication capacity [33]. When the A163S mutation was combined with the A146P mutation, a further decrease in fitness was observed (50 percent relative fitness). The T242N/G248A variant had an intermediate level of fitness (67 percent relative fitness). Interestingly, the A146P/A163S/T242N/G248A variant (49 percent relative fitness) showed no additional loss of fitness than what was observed with the A146P/A163S variant.

### ES CD8^+^ T cells suppress replication of viruses containing CTL escape mutations

**Figure 2:**
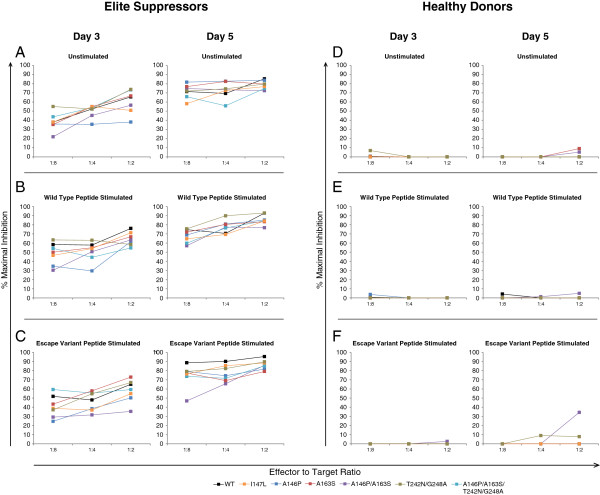
**Suppression of replication of NL4-3 escape variant viruses. A-C:** Unstimulated CD4^+^ T cells from HLA-B*5703 positive ES were infected with one of seven NL4-3 variants (wild type, black; I147L, orange; A146P, navy; A163S, red; A146P/A163S, purple; T242N/G248A, brown; A146P/A163S/T242N/G248A, teal) and cultured with autologous CD8^+^ T cells isolated from fresh PBMCs **(A)** or PBMCs stimulated with either wild type **(B)** or escape variant **(C)** HLA-B*57- Gag restricted Gag peptides for 7 days before isolation. CD8^+^ T cells were co-cultured with infected CD4^+^ T cells at three effector to target ratios. **D-F**: Unstimulated CD4^+^ T cells from healthy donors were infected with one of the seven NL4-3 variants used above and co-cultured with CD8^+^ T cells as was done with ES (**D**, unstimulated; **E**, wild type peptide stimulated; **F**, escape variant stimulated). Infection of CD4^+^ T cells was quantified by flow cytometry on days 3 (left) and 5 (right) after infection by GFP expression. Median values are plotted. For ES, n = 7. For healthy donors, n = 5.

We next determined whether CD8^+^ T cells from ES could inhibit the infection of viruses harboring escape mutations. We isolated CD4^+^ and CD8^+^ T cells from seven HLA-B*5703^+^ ES and five healthy seronegative donors. The ES did not have any other protective alleles. Unstimulated CD4^+^ T cells were infected with each variant individually and were co-cultured with unstimulated CD8^+^ T cells at various effector to target ratios for three or five days. Interestingly, unstimulated CD8^+^ T cells from ES were capable of suppressing each variant (Figure 2A), whereas healthy donors had no suppressive capabilities (Figure 2D). The percentage of suppression decreased at lower effector to target ratios. Compared to day three, day five showed increased levels of suppression at all effector to target ratios (Figure 2A).

**Figure 3:**
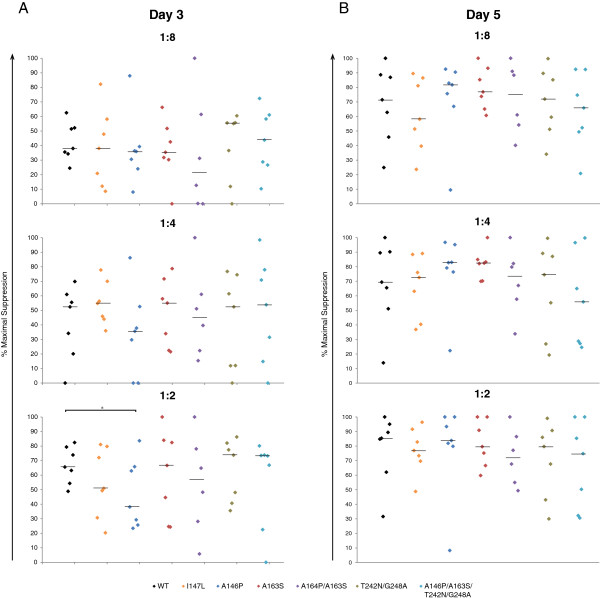
**Individual suppression of NL4-3 escape variant viruses.** Unstimulated CD4^+^ T cells from HLA-B*5703^+^ ES were infected with one of seven NL4-3 variants (wild type, black; I147L, orange; A146P, navy; A163S, red; A146P/A163S, purple; T242N/G248A, brown; A146P/A163S/T242N/G248A, teal) and cultured with autologous unstimulated CD8^+^ cells at three effector to target ratios. **A** shows maximal suppression on day 3; **B** shows maximal suppression on day 5. Black horizontal bars indicate median. Asterisk indicates P < 0.05.

A prior study demonstrated that CD8^+^ T cells that were stimulated with peptides were more effective at eliminating HIV-1 infected target cells [18]. We therefore stimulated PBMCs from the same group of ES and healthy donors with a combination of three wild type peptides (wild type IW9, wild type KF11, and wild type TW10) or a combination of three escape variant peptides (mutant IW9 (I147L), mutant KF11 (A163S), or mutant TW10 (T242N/G248A)) in the presence of 10 units/mL IL-2. After seven days of stimulation, CD8^+^ T cells from both stimulation groups were individually isolated and cultured with unstimulated CD4^+^ T cells that were infected with either wild type virus or one of the 6 escape mutants. Interestingly, there was no statistical difference in the suppressive capacity of CD8^+^ T cells that were stimulated with either wild type or escape variant peptides (Figure 2B, C and Additional file 2: Figure S2). Figure 3 shows the suppressive ability of unstimulated CD8^+^ T cells from each patient at day three and day five. Statistically significant differences in levels of suppression between wild type and escape variants were seen only for A163P at a one to two effector to target ratio (Figure 3A); this difference was not seen at any other effector to target ratios. Taken together, these results demonstrate that ES are capable of recognizing escape variant epitopes as effectively as their non-mutated counterparts.

### Stimulation of CD8^+^ T cells with wild type or escape variant peptides increases expression of interferon gamma and perforin

**Figure 4:**
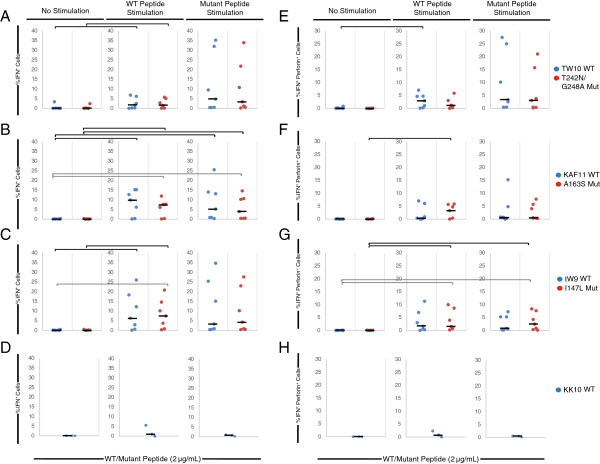
**Intracellular cytokine staining of ES CD8**^**+ **^**T cells. A-D:** CD8^+^ T cells of HLA-B*57 positive ES were either freshly isolated (left) or stimulated with either wild type (center) or escape variant (right) HLA-B*57-restricted Gag peptides for 7 days. Cells from each group underwent an overnight stimulation with individual peptides. Percentage of CD8^+^ T cells expressing IFN-γ when stimulated overnight with TW10 **(A)**, KF11 **(B)**, and IW9 **(C)** in blue, or the escape mutant variant peptide containing T242N/G248A **(A)**, A163S **(B)**, and I147L **(C)** mutations in red is shown. **E-H**: Percentage of CD8^+^ T cells expressing both IFN-γ and perforin after restimulation with TW10 **(E)**, KF11 **(F)**, and IW9 **(B)** in blue, or the escape mutant variant peptide containing T242N/G248A **(E)**, A163S **(F)**, and I147L **(G)** in red is shown. **D** and **H** show CD8^+^ T cells that express IFN-γ or co-express IFN-γ and perforin when PBMCs were stimulated overnight with Gag 263-272 (KK10, HLA-B*27^+^ peptide). Black horizontal bars indicate statistically significant difference (P < 0.05) between samples when stimulated overnight with the same variant peptide; gray horizontal bars indicate statistically significant difference (P < 0.05) between samples when stimulated overnight with opposite variant peptide.

To determine the mechanism of CTL suppression of escape mutants, we analyzed the expression of IFN-γ and perforin in freshly isolated CD8^+^ T cells and CD8^+^ T cells that were primed with either wild type or escape variant peptides. Freshly isolated PBMCs and PBMCs from each stimulation group were stimulated overnight with individual wild type or escape variant peptides. Freshly isolated CD8^+^ T cells were observed to have very low levels of IFN-γ expression in response to each peptide (Figure 4A, B, C left panel). In contrast, culture of PBMCs with wild type peptides over a 7 day period prior to overnight stimulation with wild type peptides resulted in a statistically significant increase (P < 0.05) in IFN-γ expression (Figure 4A, B, C center panel). A similar response was seen when the CD8^+^ T cells were stimulated with the analogous escape variant peptide. Interestingly, IFN-γ expression in response to restimulation with wild type or escape variant peptides individually were similar in CD8^+^ T cells that were cultured for 7 days in the presence of either wild type or escape variant peptides (Figure 4A, B, C right panel).

Because perforin expression is associated with CTL-mediated killing [18,31,46], we also examined perforin expression by stimulated CD8^+^ T cells. Low levels of perforin and IFN-γ double positive cells were observed when freshly isolated CD8^+^ T cells were stimulated with wild type or escape variant peptides (Figure 4D, E, F left panel). In contrast, CD8^+^ T cells that had been cultured for 7 days in the presence of wild type or escape variant peptide cocktails were observed to have an increase in the percentage of IFN-γ and perforin double positive CD8^+^ T cells when stimulated overnight with either wild type or escape variant peptides. Interestingly, culturing PBMCs in the presence of wild type peptides or escape variant peptide cocktails resulted in similar levels of CD8^+^ T cells that co-expressed IFN-γ and perforin in response to overnight peptide stimulation. Additionally, overnight stimulation with KK10 (Gag 263–272), an immunodominant HLA-B*27 specific peptide, resulted in no increase in IFN-γ or perforin expression, confirming the specificity of the enhanced CD8^+^ T cell responses to the HLA-B*57-restricted peptides.

**Figure 5:**
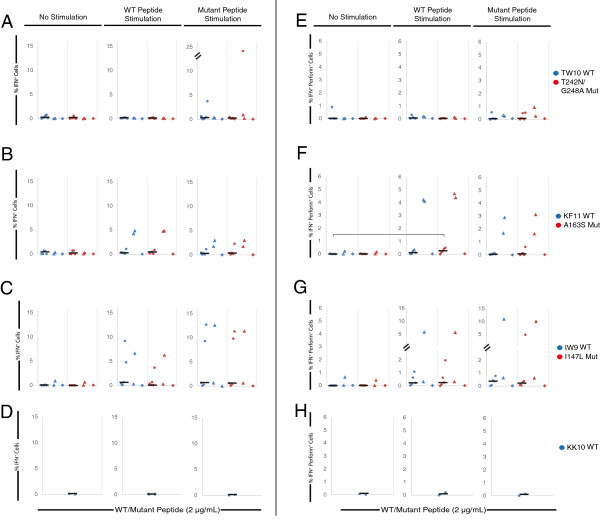
**Intracellular cytokine staining of CP CD8**^**+ **^**T cells. A-D:** CD8^+^ T cells of HLA-B*57 positive CP were either freshly isolated (left) or stimulated with either wild type (center) or escape variant (right) HLA-B*57-restricted Gag peptides for 7 days. CPs were either on suppressive HAART regiments with undetectable viral loads (circles), were on HAART regimens but recently had detectable levels of viremia (triangles), or were not on HAART and had high levels of viremia (diamond). Cells from each group underwent an overnight stimulation with individual peptides. Percentage of CD8^+^ T cells expressing IFN-γ when restimulated with TW10 **(A)**, KF11 **(B)**, and IW9 **(C)** in blue, or the escape mutant variant peptide containing T242N/G248A **(A)**, A163S **(B)**, and I147L **(C)** mutations in red is shown. **E-H**: Percentage of CD8^+^ T cells expressing both IFN-γ and perforin after overnight stimulation with TW10 **(E)**, KF11 **(F)**, and IW9 **(G)** in blue, or the escape mutant variant peptide containing T242N/G248A **(E)**, A163S **(F)**, and I147L **(G)** mutations in red is shown. **D** and **H** show CD8^+^ T cells that express IFN-γ or co-express IFN-γ and perforin when PBMCs were stimulated overnight with Gag 263-272 (KK10, HLA-B*27^+^ peptide). Black asterisks indicate statistically significant difference (P < 0.05) between samples when restimulated with the same variant peptide; gray asterisks indicates statistically significant difference (P < 0.05) between samples when restimulated with opposite variant peptide.

To determine whether similar responses were present in HLA-B*57 positive CPs, we also analyzed CD8^+^ T cells from five CPs on suppressive HAART regimens, two CPs who recently had detectable viremia, and one CP with high levels of viremia who was not on HAART. With freshly isolated PBMCs, there was very low IFN-γ expression on CD8^+^ T cells in response to overnight stimulation with peptides (Figure 5A, B, C left panel). Stimulation of cells with wild type peptides or escape variant peptides induced an increase in IFN-γ expression by CD8^+^ T cells in some patients after overnight stimulation with TW10 or KF11 (Figure 5A, B, C center and right panels). The percentage of CD8^+^ T cells that co-expressed IFN-γ and perforin increased from the unprimed baseline (Figure 5E, F, G), though generally not as dramatically as the increase seen in ES. This is consistent with the enhanced proliferative capacity of ES HIV-specific CD8^+^ T cells [18,27]. Interestingly, the patients on HAART who recently had detectable levels of viremia had higher responses than the patients on suppressive HART regimens who maintained undetectable viral loads consistent with a boosting effect of viral replication.

## Discussion

CTL responses against Gag epitopes have been associated with virologic control [47-49] and Gag-specific CD8^+^ T cells can target incoming virions and therefore have the potential to kill cells prior to productive infection [50-54]. The CTL response has been associated with the appearance of escape mutations in HIV and SIV infection. The mechanism of escape observed for individual mutations can vary. Mutations can affect epitope processing, stability of peptides, MHC:peptide complex stability, or TCR recognition of the MHC:peptide complex. Mudd *et al.* observed that Mamu-B*00801 macaques that controlled viral infection acquired few, if any, escape mutations in Vif and Nef epitopes, whereas macaques that progressed acquired several during the acute phase, suggesting that control may result from a immunologic pressure that prevents the appearance of escape mutations [55]. In contrast, Migueles *et al.* found that there was no difference between HLA-B*57^+^ CPs and HLA-B*57^+^ ES in the frequency of escape mutations in Gag [37], and Bailey *et al.* found a high frequency of escape mutations in HLA-B*57-restricted epitopes present in virus amplified from ES plasma [38,39].

In this study, we sought to determine how ES maintain control of viral replication despite circulating escape mutant viruses in the plasma. We constructed a series of mutants that contained commonly observed HLA-B*57 restricted Gag escape mutations. While our study is limited by the fact that we did not study viral inhibition of autologous escape mutants isolated from each ES, the A146P and T242N/G248A mutations in the IW9 and TW10 epitopes are commonly seen in our cohort [38]. Mutations in KF11 are rare in Clade B HIV-1 isolates, but one patient was found to have the A163S mutation and we demonstrated that this was in fact an escape mutation in a prior study [38]. In agreement with other studies [40-44], we found that some of the escape mutants we generated were detrimental to viral fitness. While attenuating escape mutations may contribute to elite control [56], viruses from CPs have been observed to have similar escape mutations, although compensatory mutations may partially restore viral fitness [57,58].

Klenerman and Zinkernagel demonstrated a limitation to the adaptive immune response: original antigenic sin [59]. In brief, when CTLs respond to an intracellular pathogen, any variant of the original pathogen elicits the activation of the original memory response, which is potentially less effective in the face of the new variant of the pathogen [60]. Allen *et al.* demonstrated in a cohort of HLA-A*11^+^ individuals that the CTL response that recognizes escape variants was incapable of recognizing the original, un-mutated variants, as these CD8^+^ T cells express unique Vβ segments [61]. New CTL responses have been shown to arise not only during the acute phase, but during chronic infection in HLA-A*02^+^ patients [62]. Lichterfeld *et al.* have shown that HLA-B*27^+^ individuals can develop a *de novo* response to the immunodominant KK10 L268M escape mutation during chronic infection [63]. In a previous study, HLA-B*57/58^+^ children infected perinatally showed a remarkable ability to generate *de novo* CD8^+^ T cell responses to escape mutations in the TW10 Gag epitope. Interestingly, there was little recognition of the wild type TW10 epitope in these children [64]. Another study found that CD8^+^ T cells from both HLA-B*57^+^ ES and HLA-B*57^+^ viremic patients made responses to autologous TW10 escape variant peptides [65]. Furthermore, we previously have described CD8^+^ T cell *de novo* responses to escape mutants in HLA-B*57^+^ ES [38,66].

While of all these studies examined IFN-γ responses, secretion of this cytokine is not a correlate of immunity in HIV infection [67,68]. Furthermore, discrepancies between IFN-γ ELISPOT assays and CD8^+^ T cell-mediated killing of both SIV and HIV escape variants have been reported [69,70]. Therefore in order to determine whether protective de novo responses were present in ES, we looked at the ability of CD8^+^ T cells to suppress replication of escape mutants. We used the suppression assay because Saez-Cirion and colleagues have demonstrated that the ability of unstimulated primary CD8^+^ T cells to inhibit viral replication correlates with elite control of HIV-1 infection [71,73], and we have recently confirmed this finding [72]. In a prior study, we demonstrated that CD8^+^ T cells from an HLA-B*57 ES suppressed multiple rare autologous TW10 escape variants by a non-cross reactive *de novo* response [74]. In the current study, we demonstrated that this phenomenon is not limited to that one ES or to rare TW10 epitopes. CD8^+^ T cells from multiple HLA-B*5703^+^ ES were able to suppress the replication of virus containing common escape mutations in all three HLA-B*5703-restricted Gag epitopes. This is probably due to the development of CD8^+^ T cells that produce perforin in response to wild type and escape variant peptides.

Interestingly, while the presence of residual intracellular concentrations of antiretroviral drugs prevented us from infecting CD4^+^ T cells and performing the suppression assay with cells from HLA-B*57^+^ CPs, we demonstrated *de novo* IFN-γ production when CD8^+^ T cells of some CPs were stimulated with variant peptides. This is consistent with an earlier study which showed that HLA-B*57^+^ CPs made IFN-γ responses to autologous TW10 variants that were as strong, if not stronger, than the responses made by HLA-B*57^+^ ES [65]. Thus the elite control of viral replication is not solely due to the ability to recognize escape mutants. Rather, our work suggests that ES maintain control of viremia in spite of virologic escape in immunodominant epitopes because they develop protective CD8^+^ T cell responses to the escape variants. In contrast, CPs generally do not develop protective CTL responses [18,27,31,46,71-73], and a study that compared HLA-B*57^+^ ES to HLA-B*57^+^ CPs found that proliferative CTL responses as well as perforin secretion in response to HIV antigens correlated strongly with elite suppression [27]. Interestingly, we show here that stimulation with wild type and escape variant peptides can induce perforin responses to both peptides in some CPs. Thus, it may be possible to immunize subjects with both wild type and escape variant peptides in order to induce protective CD8^+^ T cell responses that will prevent the emergence of common escape mutations. Taken together, it appears that ES CD8^+^ T cells may develop effective CTL suppressive responses to escape variants; these responses in addition to the reduced fitness of the escape variants, may explain how ES maintain levels of viremia in the face of virologic escape.

## Conclusion

In this study, we demonstrate the ability of CD8^+^ T cells from ES to suppress replication of viruses harboring escape mutations in HLA-B*57-restricted Gag epitopes. The reduced fitness of these escape mutants may also contribute to elite control. Additionally, protective *de novo* CD8^+^ T cell responses to both wild type and escape variant peptides could be generated in ES and some CPs by priming PBMCs with either peptide. Induction of CD8^+^ T cells that could respond to wild type virus as well as common escape mutants would be advantageous for a CTL-based vaccine.

## Methods

### Patients

The HLA-B*57^+^ patients used in the study are described in Table 1.

**Table 1:** Demographics of HLA-B*57^+^**patients in our study**

	**Age/gender**	**First HIV positive test**	**CD4 count (cells/ul)**	**Viral Load (copies/ml)**	**Recent viremia copies/ml (months ago)**	**CD4 count Pre-HAART (cells/ul)**	**Viral load Pre-HAART (copies/ml)**	**HAART**	**Years on HAART**
ES5	62/F	1990	617	<20	NA	NA	NA	-	NA
ES6	57/F	1992	675	< 20	NA	NA	NA	-	NA
ES8	61/M	2003	643	< 75	NA	NA	NA	-	NA
ES22	52/M	2009	1638	< 20	NA	NA	NA	-	NA
ES23	61/M	1985	708	< 20	NA	NA	NA	-	NA
ES24	61/M	2009	1368	<20	NA	NA	NA	-	NA
ES34	54/M	2001	425	< 75	NA	NA	NA	-	NA
CP9	48/M	1988	1310	< 20	No	155	111,089	+	8
CP10	48/M	2007	408	< 20	No	18	297,092	+	6
CP11	47/M	1998	595	<20	No	12	300,000	+	12
CP12	50/F	2012	1293	<20	No	297	9738	+	1
CP13	50/M	2012	659	< 20	No	494	30,094	+	0.5
CP14	74/M	2004	324	<20	6800 (1)	214	60,649	+	6
CP15	44/M	2001	414	95	1281 (2)	8	155,000	+	9
CP7	61/M	2001	127	227,548	NA	NA	NA	-	NA

NA: not applicable.

## Consent

All studies were approved by the Johns Hopkins Institutional Review Board. All patients and HIV negative donors provided written informed consent before participation in this study.

### Construction of escape mutant viruses

Single-round infection by pseudotyped NL4-3 virus has been previously described [75]. In brief, *eGFP* was introduced in the *env* reading frame of pNL4-3, thus creating an *env* deficient pNL4-3 that allows for analysis of infected cells by flow cytometry. Individual point mutations were introduced by site directed mutagenesis (Agilent Technologies QuikChange II kit) and primers: A146P 5′ ggcaaatggtacatcagcccatatcacctagaactttaaatgc, I147L 5′ ggtacatcaggccctatcacctagaactttaaatgcatgg, A164S 5′ ggtaaaagtagtagaagagaagtctttcagcccagaagtaatacc, and T242N/G248 5′ cccttcaggaacaaatagcgtggatgacacataatccacc followed by 5′ ggaactactagtaaccttcaggaacaaatagcgtgg. A146P/A163S was made by sequential mutagenesis. A146P/A163S/T242N/G248A was made by insertion of the digestion product of T242N/G248A with SpeI and SbfI into the A146P/A163S plasmid. These plasmids were sequence confirmed and individually cotransfected with pCI containing the III-B *env* reading frame into HEK293T cells using Lipofectamine 2000. 48 hours after transfection, supernatant was collected and virus was isolated by ultracentrifugation at 50,000 × g through a 20% sucrose cushion for 2 h.

### PBMC peptide stimulation

PBMCs were isolated from blood of ES, CP, and healthy donors by Ficoll gradient centrifugation. PBMCs were then cultured in RPMI 1640 supplemented with 10% FBS and 10 units/mL IL-2 in the presence of either 10 ug/mL total of TW10, KF11, and IW9 peptide (3 ug/mL each), or corresponding peptides containing escape mutations, for 7 days, with IL-2 supplemented every 48 h.

### CD8^+^ And CD4^+^ T cell isolation

PBMCs were isolated by Ficoll gradient centrifugation. CD8^+^ T cells were isolated from PBMC using the Miltenyi Human CD8 Microbeads according to the manufacturer’s instructions. CD4^+^ T cells were isolated from either bulk PBMCs or CD8-depleted PBMCs using the Miltenyi Human CD4^+^ T Cell Isolation Kit II according to the manufacturer’s instructions. Purity of both cell types was routinely greater than 95% as determined by staining with CD3-Pacific Blue and CD8 APC or CD4 PerCP-Cy5.5 (BD).

### Suppression

Freshly isolated CD4^+^ T cells were spinoculated as described [76] at 1,200 × *g* for 2 h with one of seven NL4-3 pseudotyped viruses in 2.9 × 10^6^ cells/tube. We typically used 50 to 100 ng p24 virus for 100,000 CD4^+^ T cells which typically resulted in 2 to 10% GFP positive cells. Cells without virus were spinoculated as a negative control. After spinoclulation, CD4^+^ T cells were washed and plated in a 96 well plate at 0.1 × 10^5^ cells / well in RPMI 1640 supplemented with 10% FBS. Unstimulated, wild type stimulated, or Mutant stimulated CD8^+^ T cells were immediately added to the spinoculated CD4^+^ T cells, at specified ratios. Cells were cultured for 3 or 5 days before fixation and staining (CD3 Pacific Blue, CD8 APC, BD) and analysis by flow cytometry on a FACSCanto II (BD).

### Intracellular cytokine analysis

0.5 × 10^6^ unstimulated or stimulated PBMCs (from above) were restimulated with peptide (2 ug/mL), anti-CD28, and anti-CD49d in the presence of GolgiStop and GolgiPlug (BD) for 12 h. After staining with CD3 PE and CD8 APC-H7 (BD), cells were fixed and permeablized with Cytoperm/Cytofix Kit (BD). Cytokines were stained using IFN-γ PerCP-Cy5.5 (BD) and Perforin FITC (Cell Sciences). Stained cells were analyzed by flow cytometry on a FACSCanto II (BD).

### Viral quantification

Concentrated virus was quantified by q-RTPCR. Viral RNA was isolated in triplicate with ZR Viral RNA Kit (Zymogen), and RT was performed using Superscript III First-Strand Synthesis Kit (Invitrogen) using poly(dT)_20_ primers. VQA qPCR was performed as described [77]. In brief, Taqman Fast Advanced Master Mix (Invitrogen) was used with primers 5′ cagatgctgcatataagcagctg and 5′ ttttttttttttttttttttttttgaagcac, and run on a ViiA7 (AB).

### Fitness assay

PBMCs were isolated from healthy donors by Ficoll gradient centrifugation and activated in RPMI 1640 supplemented with IL-2 (100 units/mL) and PHA (1 ug/mL) for 3 days. CD4^+^ T cells were isolated (as above) and spinoculated (1,200 × g, 2 h) in a 96-well plate with 0.1 × 10^6^ cells / well, with different concentrations of each virus as shown in Figure 1. 72 h after spinoculation, cells were fixed (3.3% formaldehyde) and the level of infection for each concentration of virus was analyzed by flow cytometry on a FACSCanto II (BD). GraphPad Prism was used to generate model curves. Relative fitness was determined as a percentage of the maximal infection of an individual virus relative to the wild type control.

## Competing interests

We, the authors, declare that we have no competing interests.

## Authors’ contributions

CP and RWB performed all the experiments and helped draft the manuscript. RFS participated in the study design and helped to draft the manuscript. JNB conceived of the study, participated in its design and coordination and helped to draft the manuscript. All authors read and approved the final manuscript.

## Supplementary Material

Additional file 1: Figure S1 Theoretical nonlinear regression curves plotted with each escape variant. Data generated in fitness assay is shown here (wild type, black; I147L, orange; A146P, navy; A163S, red; A146P/A163S, purple; T242N/G248A, brown; A146P/A163S/T242N/G248A, teal). Black line depicts theoretical nonlinear regression curve generated by GraphPad. R^2^ values for each theoretical curve is show on the bottom right of individual plots. Plot on the bottom right depicts all theoretical curves corresponding to NL4-3 variant color on the same plot. Click here for file

Additional file 2: Figure S2 Comparison of stimulation status for suppressive function. Suppressive capacity of CD8^+^ T cells, either unstimulated or stimulated with peptides corresponding to HLA-B*57 Gag epitope (WT) or escape mutant variant (Mutant), is compared for each of seven different escape mutant variant viruses used (wild type, black; I147L, orange; A146P, navy; A163S, red; A146P/A163S, purple; T242N/G248A, brown; A146P/A163S/T242N/G248A, teal). Suppression on day 3 (left) and day 5 (right) is shown. n=7. Click here for file
