# Supplementary figures and images for: CD8+ T cells from HLA-B*57 elite suppressors effectively suppress replication of HIV-1 escape mutants
Source: Retrovirology. 2013 Dec 12;10:152. doi: 10.1186/1742-4690-10-152 (PMC3878989; doi:10.1186/1742-4690-10-152)

**A146P**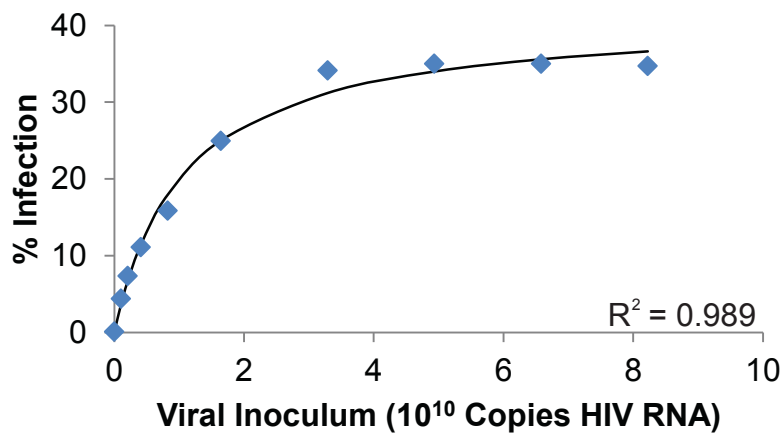**A163S**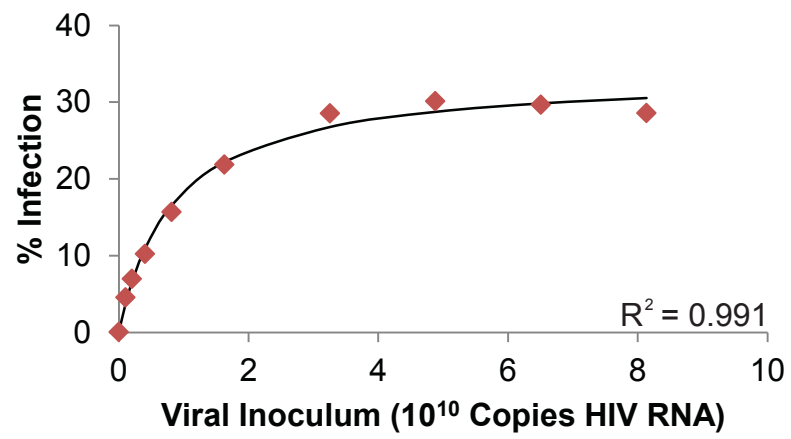**A146P/A163S**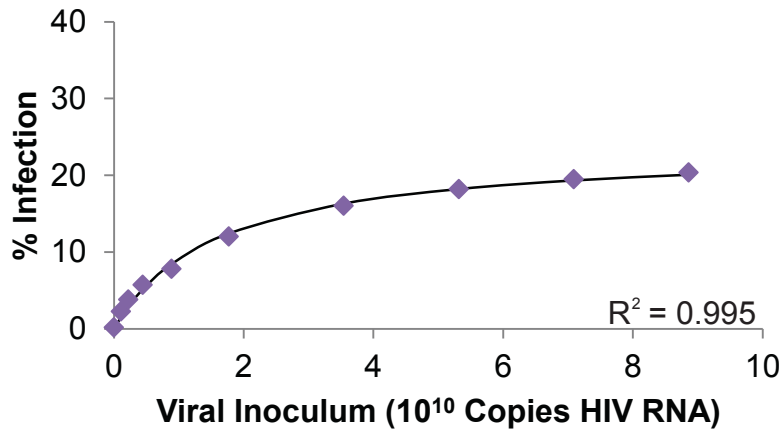**I147L**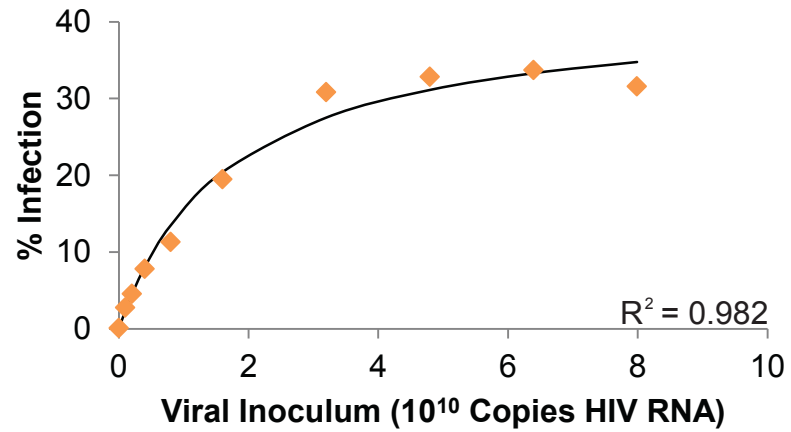**T242N/G248A**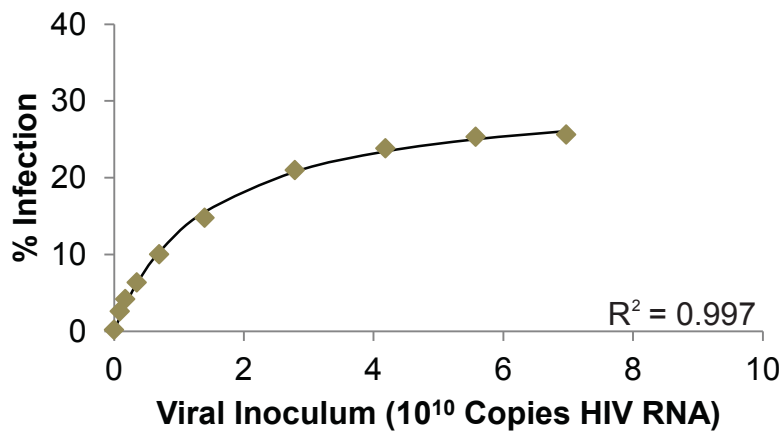**A146P/A163S/T242N/G248A**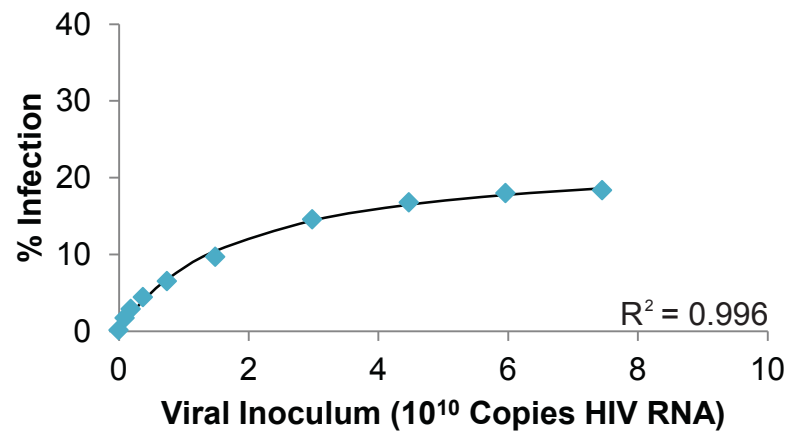**WT**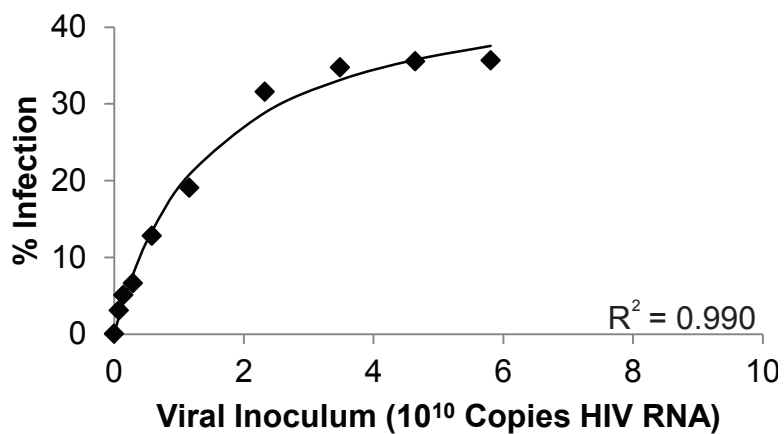**All Viruses**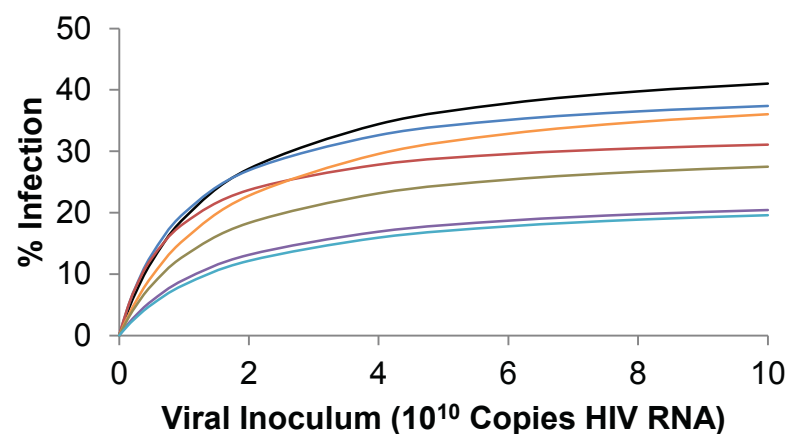

Figure S1

Supplement: Additional file 1: Figure S1 — Theoretical nonlinear regression curves plotted with each escape variant. Data generated in fitness assay is shown here (wild type, black; I147L, orange; A146P, navy; A163S, red; A146P/A163S, purple; T242N/G248A, brown; A146P/A163S/T242N/G248A, teal). Black line depicts theoretical nonlinear regression curve generated by GraphPad. R2 values for each theoretical curve is show on the bottom right of individual plots. Plot on the bottom right depicts all theoretical curves corresponding to NL4-3 variant color on the same plot. [file 1742-4690-10-152-S1.pdf]

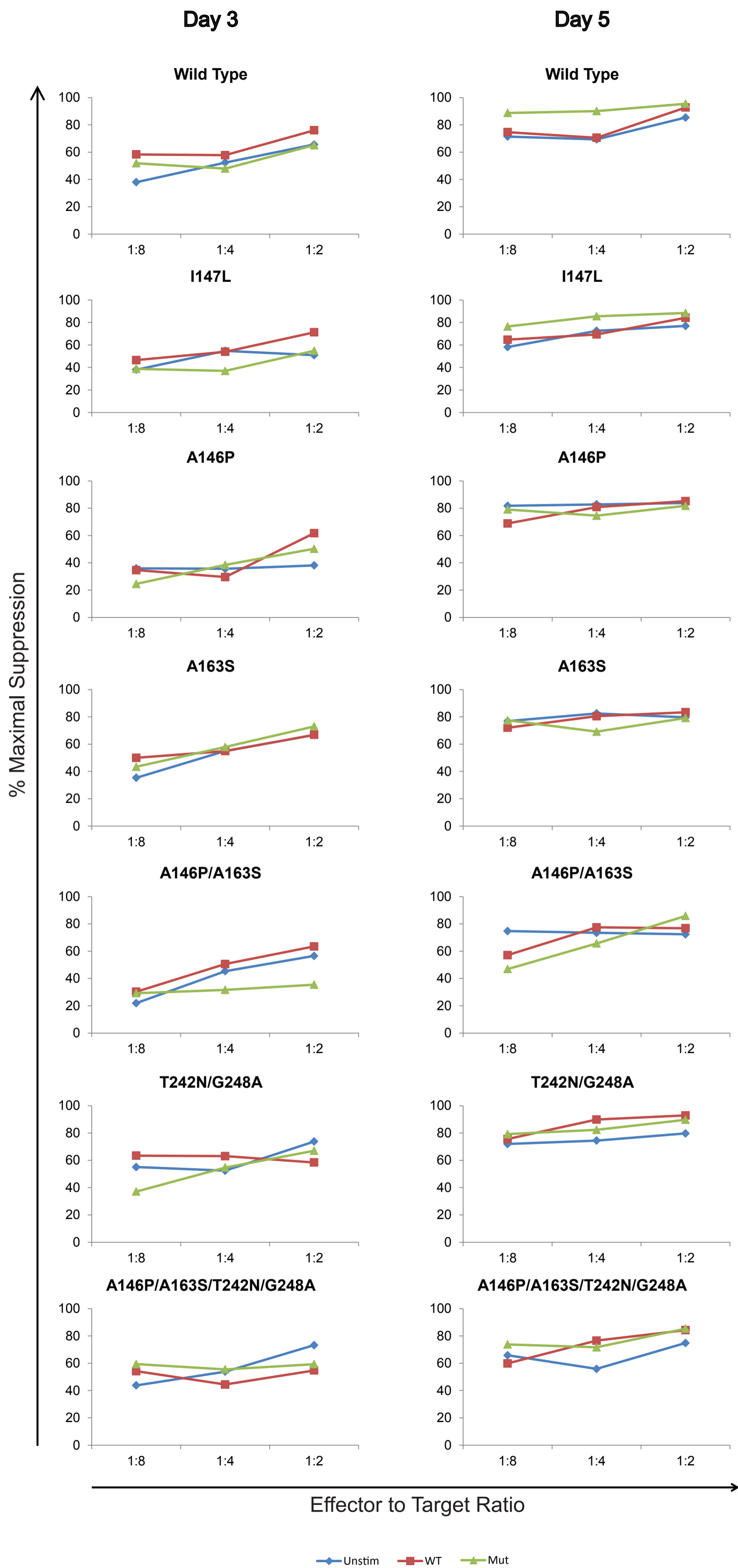

Figure S2

Supplement: Additional file 2: Figure S2 — Comparison of stimulation status for suppressive function. Suppressive capacity of CD8+ T cells, either unstimulated or stimulated with peptides corresponding to HLA-B*57 Gag epitope (WT) or escape mutant variant (Mutant), is compared for each of seven different escape mutant variant viruses used (wild type, black; I147L, orange; A146P, navy; A163S, red; A146P/A163S, purple; T242N/G248A, brown; A146P/A163S/T242N/G248A, teal). Suppression on day 3 (left) and day 5 (right) is shown. n=7. [file 1742-4690-10-152-S2.pdf]
